# Predictive modeling of brain herniation risk factors and critical thresholds in spontaneous intracerebral hemorrhage: a pilot study

**DOI:** 10.3389/fneur.2025.1612346

**Published:** 2025-11-03

**Authors:** Jinxiu Peng, Shugang Wang, Jinpeng Wang, Bing Liu, Yimei Yuan, Lishan Yin

**Affiliations:** ^1^Department of Neurosurgery, Weifang People’s Hospital, Shandong Second Medical University, Weifang, China; ^2^School of Basic Medical Sciences, Shandong Second Medical University, Weifang, China; ^3^Department of Immunology, Weifang Hospital of Traditional Chinese Medicine, Weifang, China; ^4^Department of Neurosurgery, Affiliated Hospital of Shandong Second Medical University, Weifang, China

**Keywords:** spontaneous intracerebral hemorrhage, basal ganglia/thalamic hemorrhage, brain herniation, hematoma volume, midline shift, intraventricular hemorrhage

## Abstract

**Objective:**

Patients with spontaneous intracerebral hemorrhage (sICH) could benefit from personalized treatment strategies. Developing a rational classification system is therefore essential to guide clinical decision-making. This study aimed to identify independent predictors of brain herniation in sICH patients and establish critical thresholds for these predictors, using clinical and imaging data.

**Methods:**

We retrospectively analyzed consecutive spontaneous intracerebral hemorrhage patients admitted between June 2020 and December 2023. Demographics, medical history, clinical parameters on admission, and CT findings were collected. Hematoma volume and midline shift were quantified using 3D Slicer software, while intraventricular hemorrhage was graded by the Graeb score. Brain herniation was defined by acute neurological deterioration (e.g., loss of consciousness, anisocoria, or posturing) and CT evidence of critical structural displacement (e.g., obliteration of perimesencephalic cisterns, uncal herniation). Statistical methods included receiver operating characteristic curve analysis and multivariable binary logistic regression to identify independent predictors of herniation.

**Results:**

Fifty-five patients met inclusion criteria. Among them, 58 CT scans were analyzed. Multivariable analysis identified three independent predictors of cerebral herniation in basal ganglia/thalamic hemorrhages: hematoma volume >64 mL (adjusted OR = 14.67; 95% CI: 1.44–149.82; *p* = 0.023), midline shift at the interventricular foramen >11 mm (adjusted OR = 10.05; 95% CI: 1.61–62.69; *p* = 0.014), and Graeb score (per 1-point increase: adjusted OR = 1.47; 95% CI: 1.08–2.00; *p* = 0.015).

**Conclusion:**

Among four midline structures analyzed, midline shift at the interventricular foramen was the strongest predictor. Key herniation predictors for basal ganglia/thalamic hemorrhage comprise hematoma volume >64 mL, midline shift at the interventricular foramen >11 mm, and Graeb score.

## Introduction

Spontaneous intracerebral hemorrhage (sICH) is one of the most lethal and disabling subtypes of stroke (1, 2), accounting for approximately 10% of the nearly 795,000 annual stroke cases in the United States and exhibiting even higher prevalence in low- and middle-income countries (3, 4). The aging population and widespread use of anticoagulants are likely contributing to a rising incidence of sICH (5, 6), yet treatment advancements have been limited over the past decades (6, 7). This underscores the urgent need for innovative therapies and more effective implementation of existing strategies.

The role of surgical intervention in sICH remains controversial due to inconsistent evidence. While Auer et al. (8) suggested that younger patients with smaller, subcortical hematomas may benefit most from surgery, Wang et al. (9) reported potential advantages for patients with moderate-sized basal ganglia hematomas (25–40 mL). In contrast, Mendelow et al. (10) found no significant benefit of surgical intervention over medical treatment in unselected patient populations. These conflicting findings highlight the need for personalized treatment strategies, which require a robust classification system based on key risk factors.

While indispensable for initial risk stratification in ICH, the Glasgow Coma Scale (GCS) and ICH Score offer limited utility for guiding specific surgical strategies. Specifically, the ICH Score predicts global mortality yet neglects dynamic pathological progression. Crucially, the presence of established brain herniation and the risk of imminent herniation constitute pivotal determinants of surgical decision-making—directly governing intervention urgency and modality selection (e.g., decompressive craniectomy vs. minimally invasive evacuation). To address this gap, we conducted a study to identify quantifiable thresholds for key herniation risk factors, establishing a practical stratification system for clinical decision support.

## Methods

### Study design and participants

**Figure 1:**
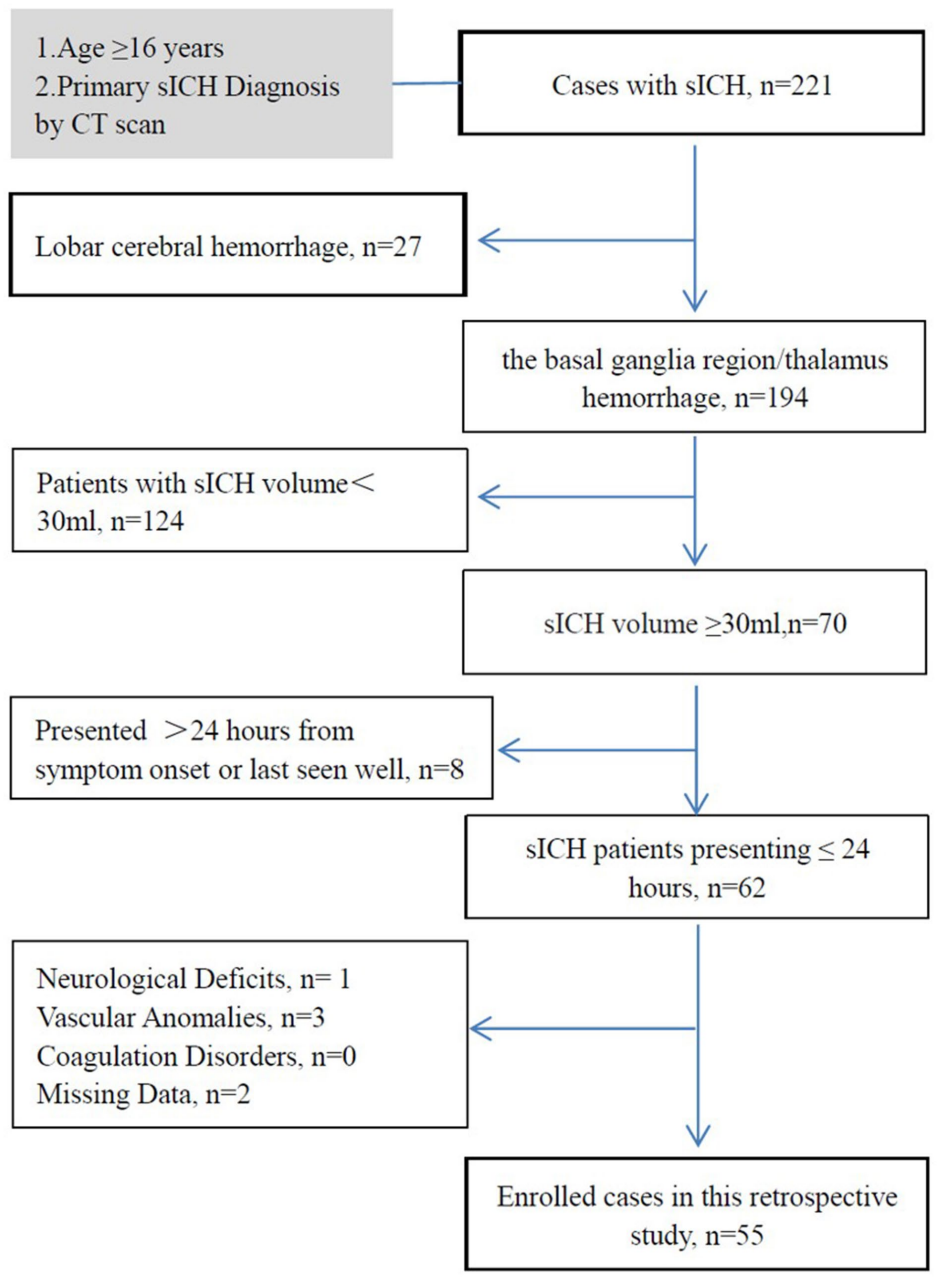
Flow diagram of study inclusion and exclusion criteria. This study investigates the patient’s condition within a set time window to determine the occurrence of brain herniation. Time window for the study: (1) for surgical patients, the study ends at the start of the surgery. (2) For patients receiving conservative treatment, the study ends at the onset of brain herniation or 24 h after the onset of symptoms. (3) If the treatment plan for a patient changes from conservative to surgical within 24 h of onset, the study ends at the start of the surgery. sICH, spontaneous intracerebral hemorrhage; CT, computed tomography.

We conducted a retrospective analysis of consecutive patients with sICH and hematoma volumes ≥30 mL in the basal ganglia or thalamus, treated between June 2020 and December 2023. The study included primary sICH patients aged ≥16 years admitted within 24 h of symptom onset or last seen in good health. Diagnosis was confirmed by brain computed tomography (CT) scans following American Stroke Association criteria (11). Exclusion criteria were: (1) lobar cerebral hemorrhage; (2) history of neurosurgical intervention, stroke, or traumatic intracranial hemorrhage with neurological deficits; (3) secondary sICH due to ruptured aneurysms, cerebral arteriovenous malformations, vascular anomalies, or Moyamoya disease; and (4) coagulation dysfunction disorders. The inclusion/exclusion process is illustrated in Figure 1.

**Figure 2:**
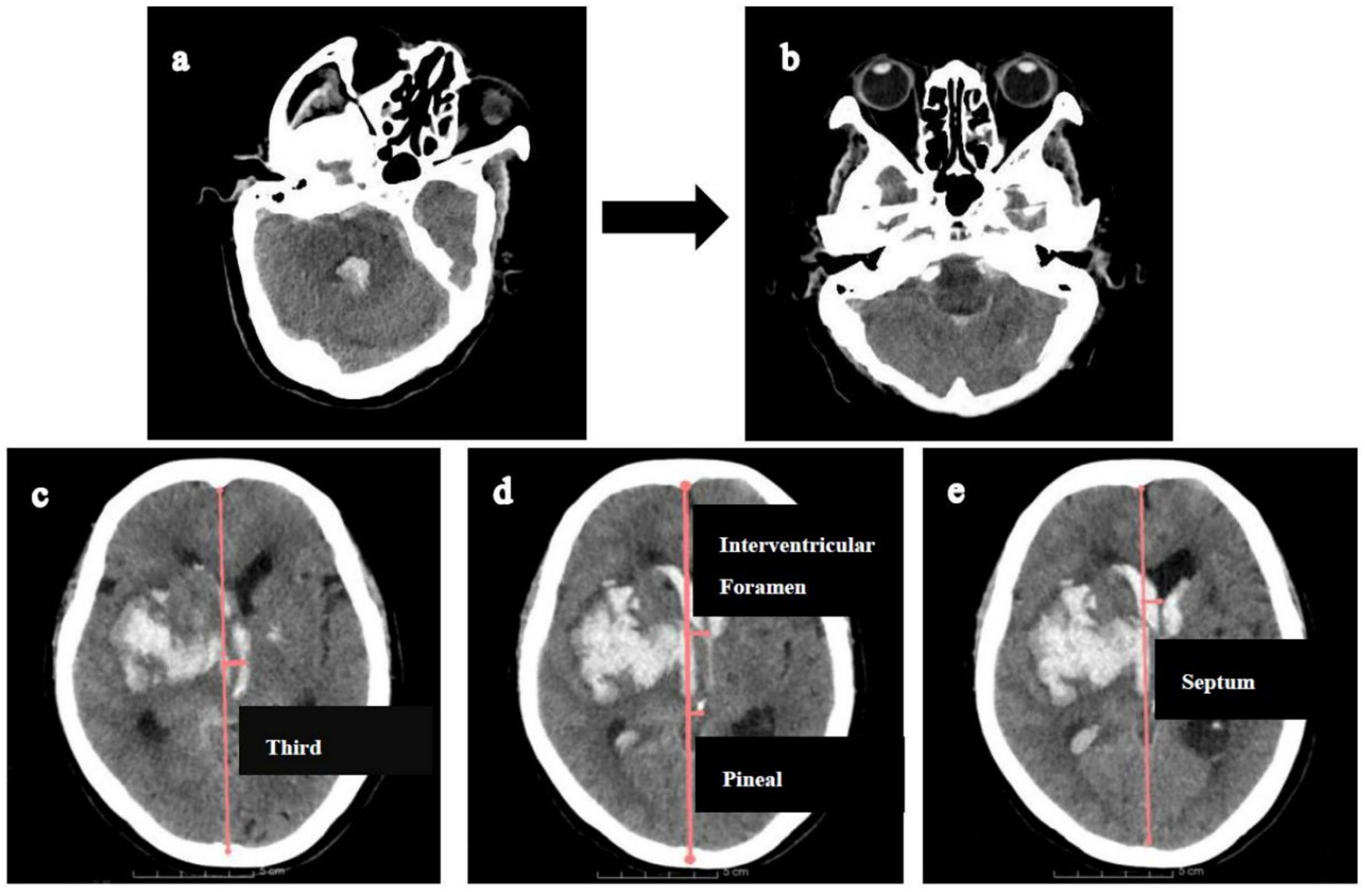
Illustration of measuring midline shifts (MLS) at various levels. Firstly, CT reconstructions **(a,b)** were aligned with the orbitomeatal line to facilitate accurate midline shift measurements. Then, MLS was quantified by constructing an ideal midline (red line), linking the most anterior and posterior visible points on the falx. **(c)**. MLS at the level of third ventricle. **(d)** MLS at the level of interventricular foramen and pineal gland. **(e)** MLS at the level of septum pellucidum.

This study adhered to the Declaration of Helsinki and was approved by the Research Ethics Committee of the Affiliated Hospital of Shandong Second Medical University (wyfy-2024-ky-173). Informed consent was obtained from all participants or legal guardians. One patient provided permission to display their CT scan demonstrating location-specific midline shifts (Figure 2).

### Management of sICH

All patients were managed according to our hospital’s stroke pathway, aligned with Chinese Stroke Association guidelines (12). A dedicated team supervised Code Stroke protocol implementation, coordinating initial evaluations, CT imaging, surgical assessment, and anesthesia preparation. After emergency treatment (including surgical interventions when indicated), patients were transferred to the neurosurgical intensive care unit. Treatment protocols targeted systolic blood pressure <140 mmHg and blood glucose ≤10 mmol/L. Post-stabilization, patients entered the stroke rehabilitation unit.

### Variables and herniation

Demographic data, medical history, admission details, laboratory results, and treatments were obtained from electronic medical records or telephone interviews. Glasgow Coma Scale (GCS) scores were categorized as: 3–8 (severe impairment), 9–12 (moderate impairment), and 13–15 (minor/no impairment).

Brain herniation was defined by (13, 14): (1) acute neurological deterioration (defined as an acute loss of consciousness, new-onset anisocoria ≥1 mm or unilateral/bilateral fixed pupillary dilation ≥5 mm with absent light reflex, or decerebrate/decorticate posturing); (2) computed tomography evidence of critical structural displacement with obliteration of perimesencephalic cisterns, uncal herniation (medial temporal lobe displacement across the tentorium), tonsillar descent below the foramen magnum, or fourth ventricular compression causing obstructive hydrocephalus.

The study assessed brain herniation within defined timeframes: (1) for surgical patients, the study ended at the start of surgery; (2) for conservatively treated patients, it ended at herniation onset or 24 h after symptom onset/last seen in good health; and (3) for patients transitioning from conservative to surgical treatment within 24 h, the study ended at surgery initiation.

### Imaging analysis

**Figure 3:**
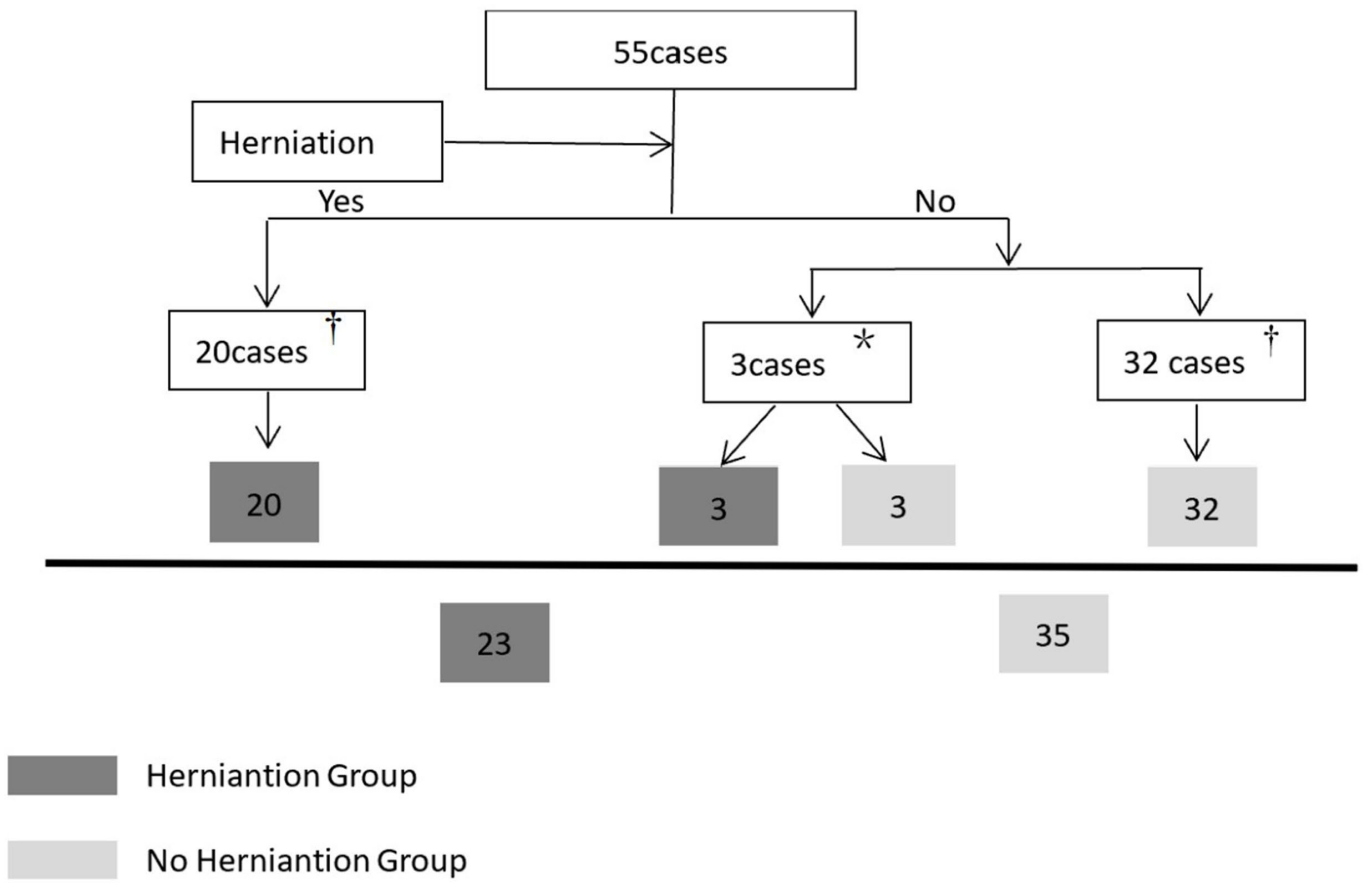
Flow diagram of collecting CT data within the defined time window. ^†^The herniation group only collects the first CT data during the herniation state, and the non-herniation group only collects the last CT data. *Three patients without initial herniation progressed to cerebral herniation within the defined time window. For the transition from a non-herniation to a herniation state: the last CT data obtained prior to herniation onset was categorized under the non-herniation group, while the first CT data captured during herniation was assigned to the herniation group. Notably, cases where hematoma volume remained unchanged were solely categorized in the herniation group.

CT data collection is illustrated in Figure 3. All brain CT images, obtained in DICOM format with a 2.5 mm slice thickness, were independently reviewed by two trained neurosurgeons. Intraventricular hemorrhage (IVH) was assessed using the Graeb score (range: 0–12), with higher scores indicating more extensive hemorrhage.

Hematoma volumes (excluding intraventricular hemorrhage) were quantified using 3D Slicer software (version 5.3.0, developed at Harvard University). Segmentation was performed in the Segment Editor module through semi-automated contouring: the Level Tracing tool delineated hematoma boundaries at 2.5-mm intervals, followed by manual refinement—adding omitted regions with the Paint tool, erasing non-hematoma inclusions. 3D reconstructions were generated from the final masks, with volumes computed via the Models module to ensure accuracy.

Midline shift quantification adhered to this protocol: (1) CT reorientation: Alignment to the orbitomeatal plane via rigid transformations (Transforms module); (2) Midline definition: an ideal midline (iML) construction connecting anterior/posterior falx cerebri dural insertions; (3) Displacement measurement: Perpendicular distances from iML recorded at: pineal gland, third ventricle, foramen of Monro, and septum pellucidum (Figure 2).

### Statistical methods

All statistical analyses were performed using IBM SPSS Statistics (version 25.0; Armonk, NY, United States) with a two-tailed alpha level of 0.05 defining statistical significance. Continuous variables were initially characterized through distributional analysis employing the Shapiro–Wilk test supplemented by quantile-quantile plot evaluation. Variables demonstrating normal distribution were expressed as mean ± standard deviation and compared between the herniation and non-herniation groups using independent Student’s *t*-tests, while non-normally distributed variables were reported as median (interquartile range) with Mann–Whitney *U* tests for intergroup comparisons. Categorical variables were summarized as frequency counts (percentages) and analyzed using Pearson’s chi-square test with Yates’ continuity correction; when expected cell frequencies were below 5, Fisher’s exact test with Monte Carlo approximation was applied.

The analytical strategy for identifying predictors and constructing the risk model followed a sequential process: First, univariable analyses were conducted to examine associations between each variable and cerebral herniation. Next, for variables exhibiting multicollinearity (defined by a variance inflation factor [VIF] > 5), ridge regression was employed to select the optimal variable based on regression coefficients, with other collinear variables excluded from further analysis. For continuous variables showing statistically significant differences in univariable analyses, receiver operating characteristic (ROC) curves were used to determine optimal cutoff points via the Youden index, and these variables were converted into dichotomous variables based on these thresholds. Variables with a *p*-value <0.20 from univariable analyses were entered into a multivariable binary logistic regression model to identify independent risk factors for herniation, using the forward likelihood ratio (Forward LR) method for variable selection. Finally, a risk prediction model was constructed based on the identified independent risk factors, and its performance was validated through assessments of discriminative ability (using ROC curves and the area under the curve [AUC]) and calibration (via the Hosmer–Lemeshow test). Results are reported as adjusted odds ratios (ORs) with 95% confidence intervals (CIs).

## Results

### Baseline characteristics of patients

During the study period, 221 consecutive sICH patients were screened, with 55 meeting inclusion criteria (Figure 1). Table 1 summarizes baseline characteristics. Twenty patients developed herniation, while 35 did not. The mean age in the herniation group (66.70 ± 12.97 years) did not differ significantly from the non-herniation group (61.23 ± 15.35 years; *p* = 0.185). Though females were more common in the herniation group (50.0% [10/20] vs. 34.3% [12/35]), this difference was non-significant (*p* = 0.252). Herniation patients had significantly lower GCS scores (*p* < 0.001). No significant differences existed in medical histories or other admission parameters (except GCS) between groups. Fifty-eight CT scans were analyzed during defined time windows (Table 2). The herniation group exhibited significantly larger hematoma volumes, higher Graeb scores, and greater midline shifts (all *p* < 0.001).

**Table 1:** Study participants’ characteristics at admission.

Variables	Herniation (*N* = 55)		*p-*value
	Yes (*N* = 20)	No (*N* = 35)	
Demographics			
Age (y), mean ± SD	66.70 ± 12.97	61.23 ± 15.35	0.185
Sex, female, *n* (%)	10(50.0%)	12(34.3%)	0.252
Medical history			
Hypertension, *n* (%)	17(85.0%)	23(65.7%)	0.122
Diabetes, *n* (%)	9(45.0%)	7(20.0%)	0.050
Prior antiplatelet or/and anticoagulant use, *n* (%)	6(30.0%)	8(22.9%)	0.559
Admission data			
Systolic BP(mmHg), mean ± SD	177.85 ± 42.59	175.71 ± 34.30	0840
Diastolic BP(mmHg), mean ± SD	100.85 ± 18.87	96.91 ± 16.17	0.418
Glasgow Coma Score			
12–15 *n* (%)	0(0.0%)	7(20.0%)	0.000
9–11 *n* (%)	0(4.2%)	12(34.3%)	
3–8 *n* (%)	20(100%)	16(45.7%)	
Left basal ganglia hemorrhage, *n* (%)	12(60.0%)	18(51.4%)	0.539
Treatment strategy			
Conservative Treatment, *n* (%)	5(25.0%)	8(22.9%)	
Minimally invasive surgery, *n* (%)	5(25.0%)	17(48.6%)	
Endoscopic surgery, *n* (%)	3(15.0%)	5(14.3%)	
Decompressive craniectomy surgery, *n* (%)	7(35.0%)	5(14.3%)	

For continuous variables, data are mean ± SD; for categorical variables, data are number (%).

SD, standard deviation.

**Table 2:** CT characteristics during defined time intervals.

CT data	Herniation (*N* = 58)		*P-*value
	Yes (*N* = 23)	No (*N* = 35)	
ICH volume (mL), mean ± SD	89.75 ± 27.53	52.51 ± 22.30	0.000
IVH, *n* (%)	22(95.7%)	21(60.0%)	0.002
IVH Graeb score, median (IQR)	6 (3–7)	1(0–3)	0.000
Midline shifts (mm)			
Interventricular foramen, mean ± SD	13.28 ± 3.97	7.00 ± 3.49	0.000
Septum pellucidum, mean ± SD	12.82 ± 4.75	6.38 ± 4.12	0.000
Third ventricle, mean ± SD	11.75 ± 3.45	6.43 ± 3.86	0.000
Pineal, mean ± SD	8.06 ± 2.68	4.29 ± 2.54	0.000

Three patients transitioned from non-herniation to herniation within the specified time window. Their last CT data before herniation were classified in the non-herniation group, and their first CT data during herniation were classified in the herniation group.

For continuous variables, data are median (IQR) or mean ± SD; for categorical variables, data are number (%).

IQR, interquartile range, SD, standard deviation.

### Analysis of sICH volume and midline shift

**Figure 4:**
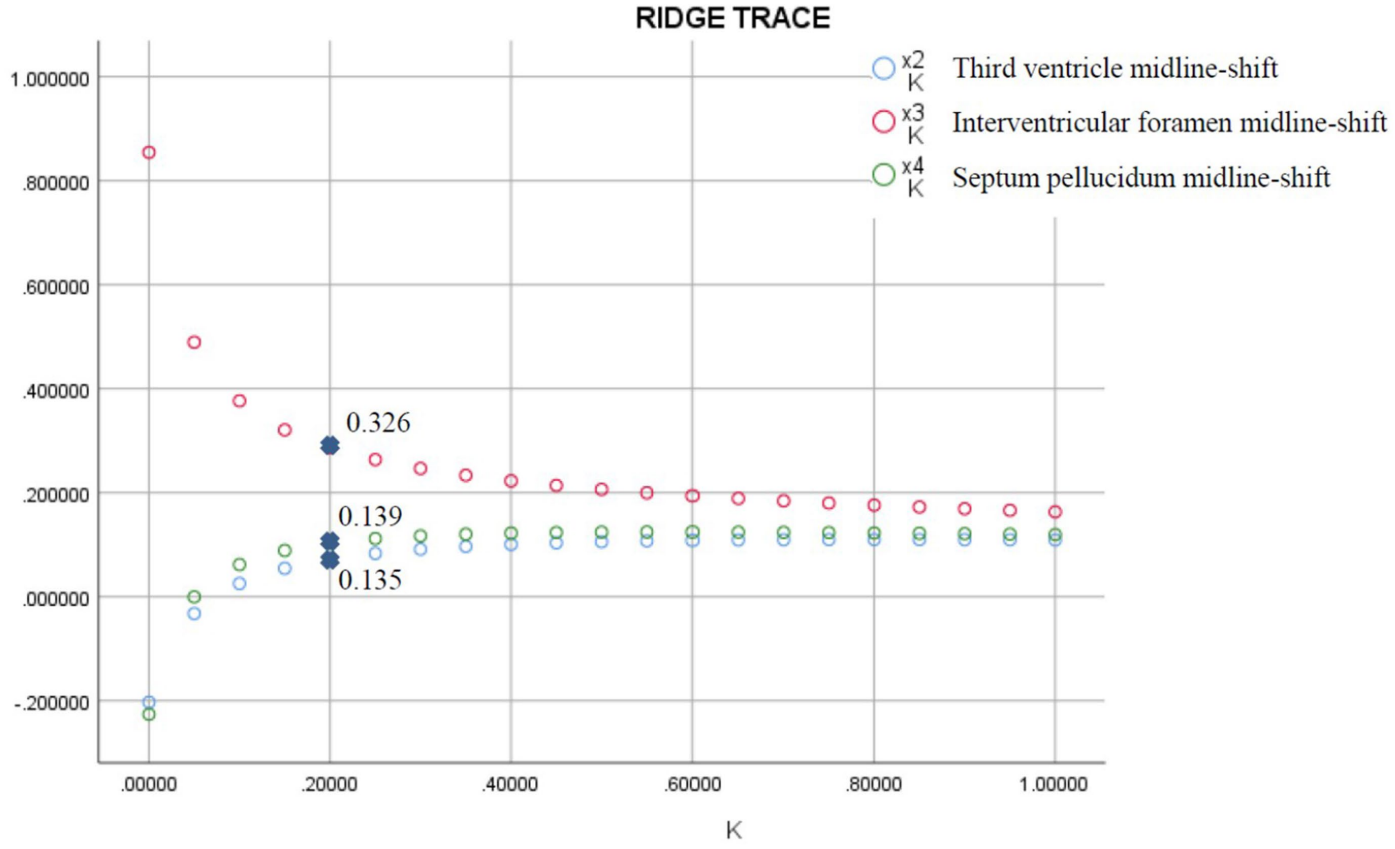
Ridge regression analysis of midline shifts for predicting brain herniation. The midline shifts of the interventricular foramen, third ventricle, and septum pellucidum exhibit multicollinearity (Table 3). Ridge regression analysis indicates that the regression coefficient for the interventricular foramen midline shifts is the largest.

Multicollinearity occurred among midline shift variables (septum pellucidum, third ventricle, interventricular foramen; Table 3). Ridge regression identified interventricular foramen shift as having the largest regression coefficient (Figure 4). Consequently, septum pellucidum and third ventricle shifts were excluded, while interventricular foramen and pineal gland shifts were retained.

**Table 3:** Collinearity statistics of midline shifts.

Variables	Tolerance	VIF
Septum pellucidum midline-shift	0.105	9.509
Third ventricle midline-shift	0.127	7.896
Interventricular foramen midline-shift	0.068	14.775
Pineal midline-shift	0.227	4.406

The shifts of the septum pellucidum, third ventricle, and interventricular foramen exhibit multicollinearity.

**Figure 5:**
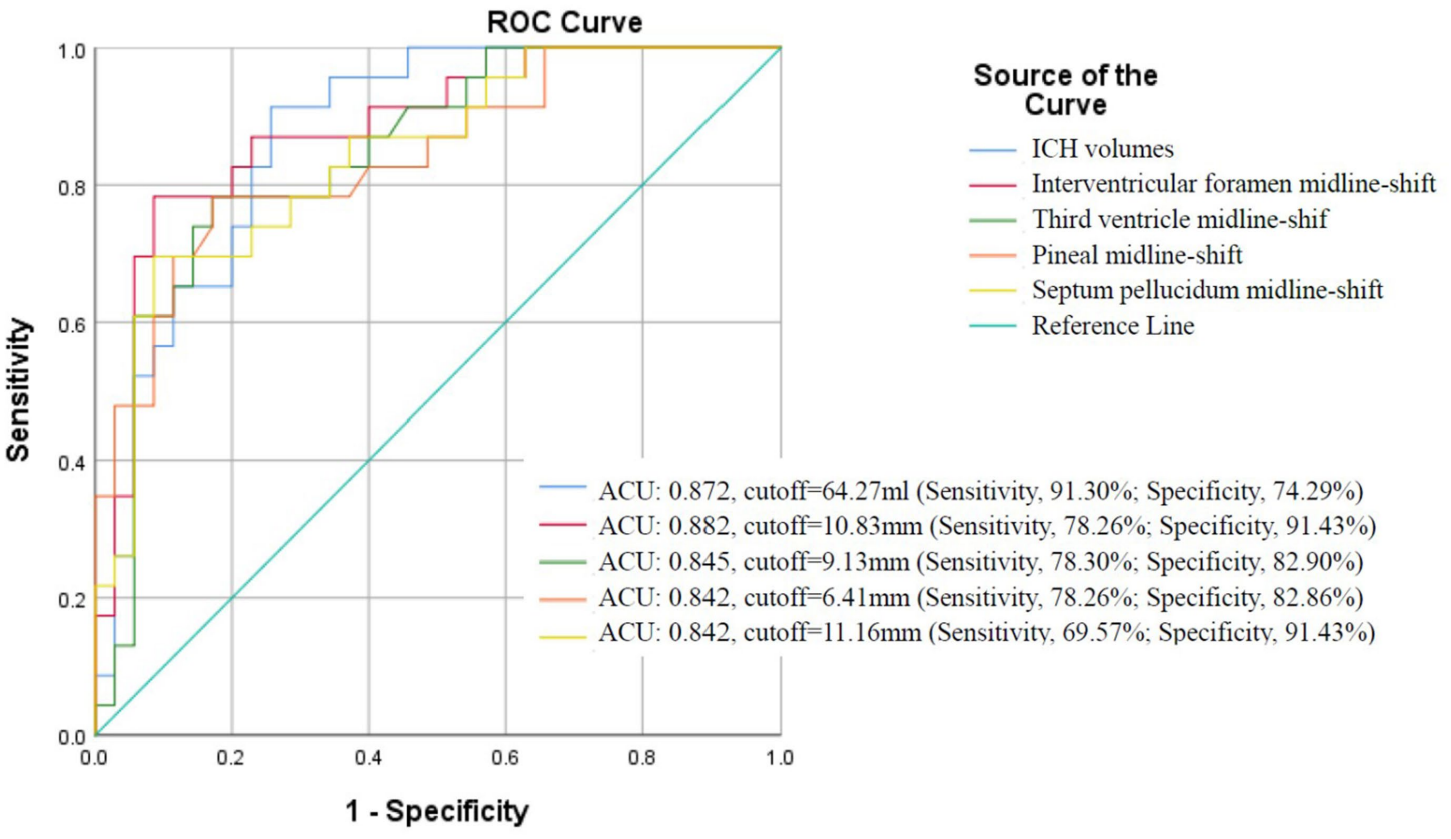
ROC curve analysis of hematoma volume and midline shifts for predicting brain herniation in sICH patients with hematoma volumes exceeding 30 mL.

The ROC analysis confirmed hematoma volume, interventricular foramen shift, and pineal gland shift as robust predictors of herniation (AUC > 0.8; Figure 5). Optimal diagnostic thresholds—hematoma volume ≥64 mL, interventricular shift ≥11 mm, and pineal shift ≥6 mm—were established through Youden index maximization. Herniation rates demonstrated significant threshold-dependent stratification: volumes ≥64 mL conferred a 67.74% herniation risk versus 7.41% for smaller volumes (*p* < 0.001); interventricular shifts ≥11 mm yielded 85.71% risk versus 13.51% (*p* < 0.001); pineal shifts ≥6 mm showed 64.29% risk versus 16.67% (*p* < 0.001).

### Brain herniation risk prediction model and validation

Forward likelihood ratio logistic regression incorporating variables with univariable associations (*p* < 0.20) identified three independent herniation predictors (all *p* < 0.05). Hematoma volume >64 mL substantially elevated risk (adjusted OR = 14.67; 95% CI: 1.44–149.82). Interventricular foramen shift >11 mm independently predicted herniation (adjusted OR = 10.05; 95% CI: 1.61–62.69), while each 1-point increase in the Graeb score was associated with a 47% higher risk (adjusted OR = 1.47 per unit; 95% CI: 1.08–2.00). Comprehensive regression outputs are detailed in Table 4.

**Table 4:** Results of the binary logistic regression model exploring the independent risk factors associated with herniation.

Variables	Variables in the equation	Herniation	
		Adjusted OR (95%CI)	*P*
sICH volume (>64.00 mL)	Yes	14.67 (1.44–149.82)	0.023
IF shift (>11.00 mm)	Yes	10.05 (1.61–62.69)	0.014
Graeb Score	Yes	1.47 (1.08–2.00)	0.015
Pineal gland shift (>6.00 mm)	No		0.882
Age (years)	No		0.704
history of hypertension (yes)	No		0.859
history of diabetes (yes)	No		0.110
Glasgow Coma Scale			
GCS 12-15 (reference)	No		0.201
GCS 9–11	No		0.140
GCS 3–8	No		0.073

Variables included in the multivariable binary logistic regression model were sICH volume cutoff, Interventricular Foramen shift cutoff, Pineal gland shift cutoff, IVH Graeb Score, age (in years), history of hypertension, history of diabetes, and Glasgow Coma Scale. Variable selection was refined using the Forward Likelihood Ratio (Forward LR) method.

OR, odds ratio; sICH, spontaneous intracerebral hemorrhage; IF, Interventricular Foramen; IVH, intraventricular hemorrhage; GCS, Glasgow Coma Scale.

**Figure 6:**
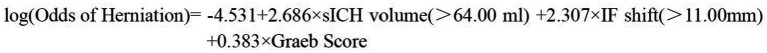
Brain herniation risk prediction model. *P* denotes the probability of herniation, and dichotomous predictors are coded as 1 if the threshold is exceeded, 0 otherwise.

**Figure 7:**
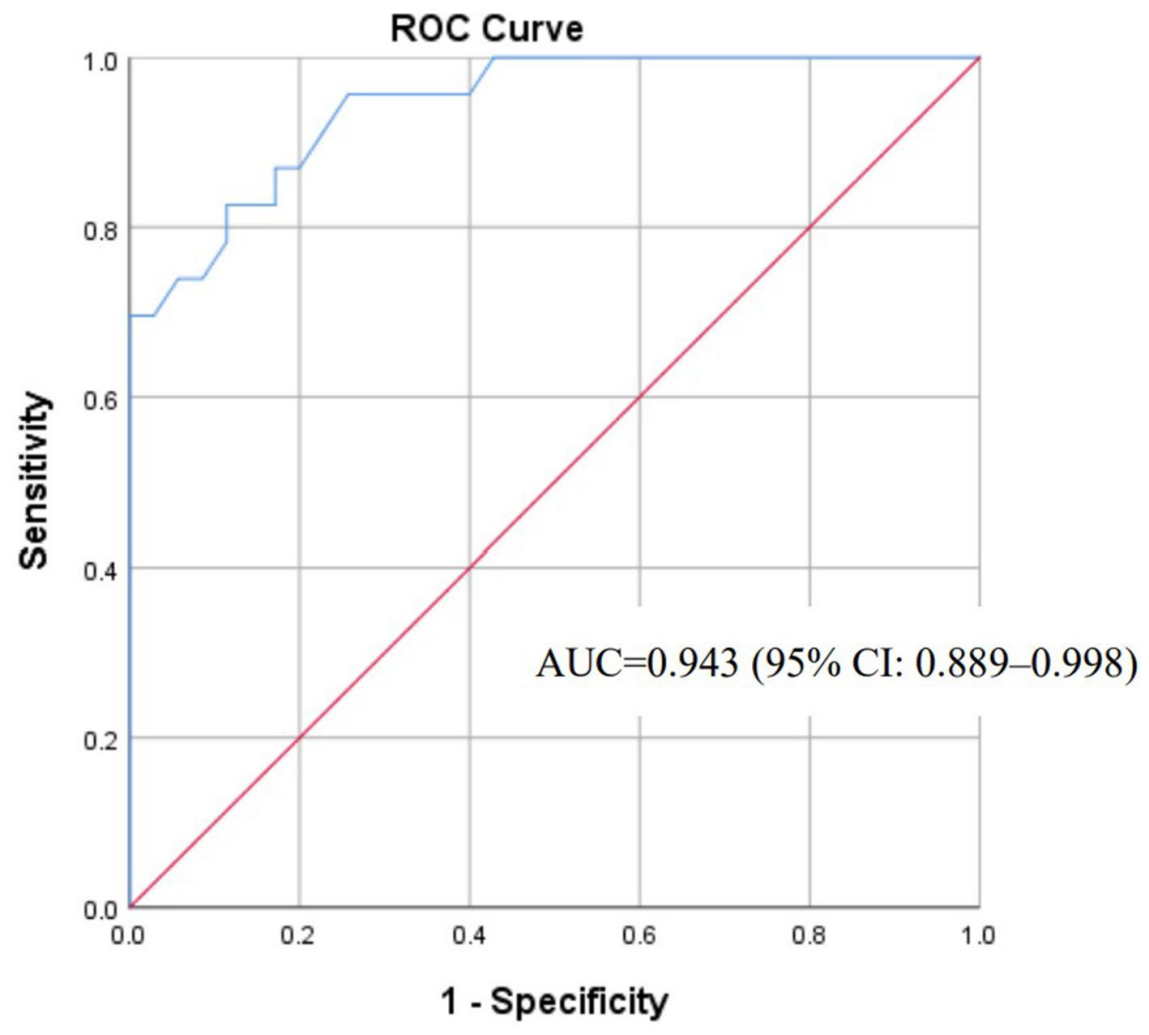
ROC curve for the brain herniation risk prediction model.

The final logistic regression equation (Figure 6) exhibited exceptional discriminative capacity (AUC = 0.943; 95% CI: 0.889–0.998; *p* < 0.001). Calibration was evidenced by a non-significant Hosmer-Lemeshow test (*χ*^2^ = 4.794, *df* = 6, *p* = 0.570). For clinical implementation, a screening probability threshold of ≥0.518 achieved balanced accuracy (sensitivity 82.6%, specificity 88.6%) for ruling out impending herniation (Figure 7).

## Discussion

Brain herniation represents a critical neurosurgical emergency in sICH, substantially worsening neurological outcomes and complicating therapeutic decision-making. Timely intervention prior to herniation onset is paramount for optimizing functional recovery, necessitating individualized risk stratification to guide surgical strategy selection. Although advanced methodologies like machine learning (ML) demonstrate increasing proficiency in predicting complex sICH outcomes—such as hematoma recurrence risk (15) or medication effect associations (16)—the precise quantification of key predictors and their clinically actionable thresholds remains foundational. Such empirically derived thresholds are indispensable both for direct bedside risk stratification and as objectively defined inputs for developing robust ML predictive models. Current research, however, has predominantly focused on identifying risk factors *per se* without establishing quantitative boundaries distinguishing high- and low-risk cohorts. This gap impedes standardized clinical decision-making and underscores the urgent need for validated tools defining actionable risk thresholds to guide tailored interventions (e.g., minimally invasive evacuation vs. medical management) and optimize resource allocation.

To address this unmet need, our study systematically evaluated clinical and neuroimaging biomarkers to identify core predictors of impending herniation. By validating critical cutoff thresholds (hematoma volume ≥64 mL, interventricular foramen shift ≥11 mm) and developing a multivariate risk prediction model, we provide clinicians with quantitative tools for dynamic risk stratification. This enables real-time classification of patients into discrete risk categories, informing escalation protocols for high-risk cases. Furthermore, these thresholds serve as rigorously defined feature inputs for future ML-based predictive systems in sICH, bridging the gap between theoretical risk association and clinical deployability.

### Hematoma volume as a predictor of brain herniation in sICH: establishing critical thresholds for clinical intervention

Hematoma volume is a pivotal prognostic factor in sICH, serving as a key determinant of intracranial pressure dynamics and neurosurgical decision-making. Consistent with established guidelines (11, 17), we set an inclusion criterion of hematoma volume ≥30 mL for basal ganglia/thalamic hemorrhages.

Cohort analysis revealed striking volumetric differences: the herniation group had significantly higher mean volumes (89.75 ± 27.53 mL) versus the non-herniation group (52.51 ± 22.30 mL; *p* < 0.001). ROC analysis identified 64 mL as a critical threshold, with herniation rates diverging dramatically below (7.41%) and above (67.74%) this cutoff. Volumes exceeding 64 mL conferred a 14.67-fold increased herniation risk (95% CI: 1.44–149.82; *p* = 0.023).

Building on these findings, we propose a novel classification system wherein basal ganglia/thalamic hemorrhages are categorized as Moderate sICH (30–64 mL) or Large sICH (>64 mL). This volumetric stratification aligns with prior observations by Moussa et al. (18), who designated hemorrhages exceeding 60 mL as “Large sICH” without pathophysiological justification for that threshold. Notably, our cutoff provides clinically actionable guidance absent in previous reports.

Based on the 2022 American guidelines for the management of sICH (11) and our findings, we empirically introduce that minimally invasive hematoma evacuation may be more suitable for the moderate sICH with volume of 30.00–64.00 mL, and either craniotomy or decompressive craniectomy for the large sICH with volume of ≥64.00 mL.

### Midline shift as a predictor of herniation in sICH: standardized measurements and clinical implications

Midline shift (MLS) is a critical prognostic indicator of mass effect. We systematically quantified MLS at four landmarks: interventricular foramen, pineal gland, third ventricle, and septum pellucidum. To enhance precision, we implemented a standardized 3D Slicer protocol: aligning bilateral external auditory canals and outer canthi on axial CT planes minimized positional artifacts. Herniation patients exhibited significantly greater MLS at all sites (Table 2), corroborating Cordonnier et al. who documented smaller MLS in comatose patients without anisocoria (19).

Although no universal consensus exists regarding optimal measurement sites for MLS in sICH, our comparative analysis identified interventricular foramen shift as a robust predictor of cerebral herniation. While traumatic ICH management employs a standardized 5-mm MLS threshold for surgical intervention (20), the absence of validated thresholds for sICH has compelled clinicians to extrapolate trauma criteria to sICH populations—an approach lacking pathophysiological justification.

To address this critical gap, our ROC analysis defined a distinct 11-mm threshold for interventricular foramen shift, revealing a dramatic escalation in herniation risk: rates increased from 13.51% (<11 mm) to 85.71% (≥11 mm). Multivariable regression further confirmed its independent association with herniation (adjusted OR = 10.05; 95% CI: 1.61–62.69; *p* = 0.014), thus offering an evidence-based quantitative standard for sICH-specific surgical decision-making. Crucially, our findings not only define this evidence-based quantitative threshold but also specify that midline shift measurement for sICH-related herniation risk must be performed at the level of the interventricular foramen, thereby providing a targeted standard for surgical decision-making.

Based on our single-center experience, we recommend that for sICH with hematoma volume exceeding 30 mL, minimally invasive hematoma evacuation may be prioritized for cases presenting midline shifts below 11.0 mm, whereas craniotomy or decompressive craniectomy should be considered for those with larger shifts.

### Intraventricular hemorrhage as a predictor of brain herniation in sICH

Intraventricular hemorrhage (IVH) complicates 30–50% of sICH cases (11, 21, 22), often precipitating acute obstructive hydrocephalus via ventricular obstruction or chronic hydrocephalus via CSF pathway dysfunction. In our cohort, 73.18% (43/58) developed IVH—exceeding population averages due to our ≥30 mL hematoma volume inclusion threshold (mean volume: 67.28 ± 30.44 mL). This aligns with established dose–response relationships where each 1 mL hematoma increase elevates IVH odds by 4% (OR = 1.04; 95% CI: 1.01–1.08) (23).

Graeb scoring quantified IVH severity: herniation patients had substantially higher median scores versus non-herniation (6 [IQR 3–7] vs. 1 [IQR 0–3]; *p* < 0.001). Multivariable analysis revealed a graded association—each 1-point Graeb increase conferred 47% greater herniation odds (adjusted OR = 1.47; 95% CI: 1.08–2.00; *p* = 0.015). These findings corroborate prior evidence linking IVH burden to worsened neurological outcomes (19, 24), while establishing its specific role in herniation pathophysiology.

### Brain herniation risk prediction model

Our prediction model integrates three key biomarkers—hematoma volume, interventricular foramen displacement, and Graeb score—to quantify herniation risk in basal ganglia/thalamic sICH. This addresses a critical gap where current practice relies on qualitative metrics (e.g., GCS, ICH Score) lacking herniation specificity.

With an AUC of 0.943, the model demonstrates excellent discrimination. Its calibration (Hosmer-Lemeshow *p* = 0.570) ensures reliable probability estimates for shared decision-making. For example, an 88% predicted risk warrants urgent decompression, while 5% risk supports conservative management.

This model’s generalizability is limited by its single-center, retrospective design and small sample (55 patients). External validation in multi-center cohorts is needed. Despite this, the model provides a framework for personalized triage, potentially reducing overtreatment and undertreatment in sICH.

## Conclusion

This study comprehensively analyzed diverse clinical and imaging indicators to identify reliable predictors of brain herniation in sICH. Our findings establish hematoma volume >64 mL, interventricular foramen shift >11 mm, and increasing Graeb score as critical risk thresholds predictive of impending herniation. Building upon these biomarkers, we developed and validated a multivariable risk prediction model demonstrating excellent discriminative capacity (AUC = 0.943) and calibration. This tool provides quantitative guidance for clinical decision-making—enabling conservative management in low-risk patients while prioritizing urgent surgical intervention for high-risk cases. Future validation in larger multicenter cohorts and integration with dynamic imaging biomarkers or machine learning approaches will further refine its clinical utility.

## Limitations

The retrospective design introduces potential selection bias and limits causal inference. Although standardized protocols using 3D Slicer software and blinded neurosurgeon assessments were implemented to ensure measurement rigor, the modest sample size (*n* = 55) constrains statistical power and generalizability. Residual confounding from unmeasured variables may persist despite multivariate adjustment. Prospective multicenter studies with larger cohorts are needed to validate these thresholds and enhance model generalizability.

Our inclusion criterion (hematoma volume ≥30 mL) intentionally excluded smaller hemorrhages with lower herniation risk. While this enhances specificity for studying herniation mechanisms, it limits generalizability to all ICH presentations. This selection bias reflects our focus on high-risk scenarios where surgical decisions are most contentious. However, it means our model may not apply to mild cases (<30 mL) where herniation risk is minimal. Future studies should validate these thresholds in broader populations, including lobar hemorrhages and smaller hematomas.

The treatment recommendations proposed in this study are empirically derived from our institutional experience and retrospective data. These suggestions should be interpreted as preliminary and hypothesis-generating rather than as established clinical guidelines. It is important to emphasize that these strategies have not been evaluated in randomized controlled trials and may be influenced by selection bias and unmeasured confounders.

## Data Availability

The raw data supporting the conclusions of this article will be made available by the authors, without undue reservation.
